# Associations between refugee camp living and duration lived in refugee camps with health outcomes: A cross-sectional analysis of the Annual Survey of Refugees, 2021–2022

**DOI:** 10.1371/journal.pone.0327608

**Published:** 2025-12-08

**Authors:** Kiran Thapa, Lana Sabbah, Rawan AlHeresh, Altaf Saadi

**Affiliations:** 1 Department of Neurology, Massachusetts General Hospital, Boston, Massachusetts, United States of America; 2 Havard Medical School, Boston, Massachusetts, United States of America; 3 Occupational Therapy Department, Faculty of Rehabilitation Sciences & Public Health Institute, University of Jordan, Amman, Jordan; Ankara University: Ankara Universitesi, TÜRKIYE

## Abstract

**Introduction:**

Refugees represent a growing, marginalized population who experience significant health disparities. Approximate 20% of refugees live in refugee camps. Quantitative studies examining the health effects of refugees living in refugee camps are limited. We examined whether living in camps (and duration) is associated with worse health among U.S. refugees.

**Methods:**

We used two years of cross-sectional data from the Annual Survey of Refugees (ASR 2021 and 2022), involving refugees ≥ 16 years old who entered the U.S. between FY 2016 and FY 2021. We tested for associations of living in a refugee camp (and duration) with self-reported physical and mental health using separate logistic regression models.

**Results:**

In this national sample of 3,005 refugees (mean age = 39.0 years, SD = 12.4 years; 46% women, 30% White, 31% Black, 18% Asian), more than one in three (37%) reported living in a refugee camp – of whom, over 88% lived in a refugee camp for a year or more or their whole life. Living in refugee camps was highest for refugees from Democratic Republic of Congo (75%), Somalia (58%), and Burma (44%). In adjusted analyses, compared to those who did not live in camps, those who lived in camps for ≥1 year had 27% greater odds of poor physical health (aOR: 1.27 [95% CI: 1.02, 1.60]). Association of camp living with mental health became insignificant when adjusted for socio-demographic characteristics.

**Conclusions:**

Refugees who lived in refugee camps, and for longer duration, may require targeted interventions to mitigate health harms from their refugee camp experience.

## Introduction

Since the passage of the Refugee Act of 1980, more than 3 million refugees from different regions of the world have been resettled into the United States (U.S.), having fled persecution in their home countries. Between 2014 and 2023, over 65% of resettled refugees came from the Democratic Republic of the Congo, Burma, Iraq, Syria, and Somalia [1]. Refugees experience several negative exposures along the displacement continuum. These include violence, conflict, trauma, and poverty in their home countries; exploitation, smuggling networks, extreme weather, and harsh travel routes during migration [2], and continued challenges after resettlement, including legal uncertainty, detention, and limited access to stable employment and housing [3].

For many, the migration journey can be protracted and arduous, involving multiple relocations or displacements, whether in refugee camps or urban settlements. Refugee camps frequently lack standard living conditions, are overcrowded, and provide limited access to essential health and other services, including clean water and sanitation [4]. These experiences can have a detrimental impact on the physical and mental health outcomes of refugees. While some may spend the majority or entirety of their lives in camp settings, others may reside in temporary facilities, such as makeshift camps in the U.S. southwest border, awaiting asylum processing.

Approximately 1 in 5 refugees live in refugee camps worldwide [5]. Refugees’ living conditions portend poor health outcomes across the displacement continuum. Studies have suggested that the higher prevalence of mental and physical health problems among refugees compared to the general population could be mostly attributed to their pre-migration trauma and migration-related stressors. Limited studies have reported a cumulative negative effect on mental health associated with the length of time spent waiting in asylum adjudication procedures, whether in refugee camps, detention, or other institutional facilities. A study in Moria camp, Greece, where refugees reside until their cases are adjudicated (average duration of stay – 71 days), found that length of stay was positively associated with acute mental health crises. Unlike refugees who live in urban areas that may provide opportunity for autonomy and employment, those living in camps may face poor housing conditions [6], geographic barriers that limit access to health care facilities [6], disconnection from community and social isolation [7], racism and discrimination [7], and fear of interacting with law enforcement authorities in health care spaces, lack of vaccination initiatives, poor sanitation and hygiene [6], food insecurity, and overcrowding [6]. These risk factors could lead to poor mental health outcomes such as anxiety and depression [8], or physical health outcomes such as respiratory illnesses, gastrointestinal issues, chronic pain, diabetes, and hypertension [9]. Chronic illnesses significantly decrease quality of life (QOL), with adverse effects persisting even after five years following displacement in refugee camps [10].

However, quantitative research is limited on whether health outcomes vary among those who lived in camps versus those who did not. Urban refugees generally report better physical health, higher satisfaction with their surroundings, and greater feelings of safety than those in camps [11]. But they also show a higher prevalence of chronic diseases such as obesity, diabetes, and hypertension [12]. Mental health outcomes vary, with camp-based refugees experiencing more post-migration stressors but fewer pre-migration traumas, while urban refugees report higher rates of PTSD and depression [13]. Examining disparities in health and well-being of refugees based on their living environments, such as camp experience, is important because it can inform and/or prioritize targeted interventions. This study seeks to fill this gap by empirically examining the association of camp living with health outcomes. Our specific objectives were to: 1) assess whether living in camps is linked to poor physical and mental health in a large, national sample of recently resettled U.S. refugees, and 2) evaluate whether camp duration influences these outcomes.

## Methods

### Study and sample design

This study is a secondary analysis of the Annual Survey of Refugees (ASR), a cross-sectional survey conducted annually by the Office of Refugee Resettlement (ORR). The ASR is the only national survey that collects data on refugees resettled in the U.S. within the last five years. The ASR sample is drawn from administrative records in the ORR’s Refugee Arrivals Data System (RADS), focusing on principal applicants (PAs) whose refugee case is the basis for admission. The individuals within the refugee households are selected for participation using a stratified probability sampling. It is designed to include an equal number of household interviews from three arrival cohorts: the most recent year, the two preceding years, and the two years before that. Further stratification is based on arrival year, region of origin, native language, age group, gender, and family size at the time of arrival. The survey is administered in 20 languages, covering about 75% of all adult refugee arrivals during the survey period. More information about the survey methodology is available elsewhere [14]. We pooled data from two annual surveys (ASR 2021 and ASR 2022) that included question about refugee camp living and duration. The eligibility criteria included refugees ≥ 16 years old at the time of the interview and who entered the U.S. between FY 2016 and FY 2021, inclusive. Only the principal applicant was asked about living in a refugee camp (N = 3,005). About 20% of participants (N = 596) reported not knowing their response or refused to answer or had a missing response for at least one of the study variables. Compared to the included sample, those excluded were more likely to be older, Black, without education, and have poor English proficiency (S1 Table). This study was exempt because we used de-identified publicly available data.

### Measures

#### Dependent Variables (Outcomes).

Our dependent variables included two self-rated health outcome measures – physical health and mental health – measured originally on a Likert scale. (Table 1). For physical health, we created a binary variable with Fair/Poor health (poor) versus Excellent/Very good/Good health (good). For mental health, we similarly created a binary variable with All the time/Most of the time/Some of the time (poor) versus A little of the time/None of the time (good).

**Table 1:** Study measures and their categorizations.

Dependent variables (Outcomes)	Question and response options	Categorization
Subjective physical health	Would you say that in general your physical health is: i) Excellent, ii) Very good, iii) Good, iv) Fair, and v) Poor	0 = Excellent/Very good/Good 1 = Fair/Poor
Subjective mental health	During the past 30 days, how often did you feel so sad that nothing could cheer you up: i) All the time, ii) Most of the time, iii) Some of the time, iv) A little of the time, and v) None of the time	0 = A little of the time/None of the time 1 = All the time/Most of the time/Some of the time
**Independent variables**	**Question and response options**	**Categorization**
Living in a refugee camp	Just before coming to the U.S., were you living in a refugee camp? i) Yes, ii) No	0 = No 1 = Yes
Duration of living in a refugee camp	How long did you live in a refugee camp? i) Your whole life, ii) Less than a year, iii) A year or more	0 = Not lived in a refugee camp 1 = Less than a year 2 = A year or more/Your whole life
**Covariates**	**Categorizations**	
Age	Continuous, in years	
Sex	0 = Male, 1 = Female	
Country of origin	14 specific countries and an unspecified ‘Other’ category. Countries with <1% of the representation in the analytic sample (Malaysia, Rwanda, and Thailand) were recoded into an “Other” category, resulting in 12 categories	
Race/Ethnicity	Asian, Black, Hispanic, Middle Eastern or North African, Mixed, White, and Other	
Highest education at arrival	None, Primary school, Middle school, High school, Higher (Some university classes, University degree, Advanced degree), Other (Technical/vocational school, religious school, Other).	
English language proficiency	0 = very well/well and 1 = poor/not at all	
Marital status	0 = Divorced or separated/Never married/Widowed/Other, 1 = Now married, spouse living in household/Now married, spouse not living in household	
Year of arrival	2016–2021	
Employment status	0 = Currently employed, 1 = Not currently employed	
Region of resettlement	Midwest, Northeast, South, and West	
Healthcare coverage	Number of months in the past year with health insurance or health care coverage (0 = All 12 months, 1 = Less than 12 months)	

#### Independent Variables.

Our independent variable of interest included i) whether refugee lived in a refugee camp (yes/no), and ii) duration of living in a refugee camp (Not lived in a refugee camp/Less than a year/More than a year or whole life) (Table 1).

### Socio-demographic covariates

We included age, gender, country of origin, race/ethnicity, marital status, highest education attainment at arrival, current English proficiency, year of arrival, employment status, resettlement region, and healthcare coverage in the past year as potential confounders in regression analyses (Table 1). Prior studies have shown that these factors are important determinants of health among refugees, which could affect exposure to pre- and post-migration stressors, access to care, and discriminatory experiences [15–18]. In addition, these factors may also influence camp living conditions and duration due to geopolitical and regional differences in conflict and displacement patterns, asylum policies, and resettlement prioritization [19,20].

### Statistical analyses

We began with descriptive statistics, comparing the socio-demographic characteristics of those who lived in a refugee camp and those who did not, using t-tests and χ2 tests. We then plotted the distribution of refugees who lived in a refugee camp by country of origin using a bar graph. For the first objective, we tested the associations between living in a refugee camp and refugee mental and physical health using separate logistic regression models. For the second objective, we repeated the logistic regression models using the duration of living in a refugee camp as our independent variable. Unadjusted and adjusted odds ratios, 95% confidence intervals, and P-values associated with living in a refugee camp and duration lived in refugee camp were estimated. Those who did not live in a camp were used as the reference group. Multicollinearity analysis revealed high collinearity between country of origin and race (variance inflation factor>5), and therefore, we only included country of origin in our multiple regression models. About 20% of the study sample had missing information on at least one of the variables of interest (S1 Table). To account for this missingness, we used multiple imputation by chained equations (MICE), incorporating all variables, to generate 20 imputed datasets with five iterations per dataset. Continuous variables were imputed via predictive mean matching, while categorical variables were imputed using logistic or polytomous regression methods, as appropriate. Parameter estimates from each imputed dataset were pooled using Rubin’s rules. A two-sided P-value less than 0.05 was considered statistically significant. Data analyses were conducted in R version 4.4.1.

## Results

### Participant characteristics

In this national sample of 3,005 refugees, the mean age was 39.0 years (SD = 12.4 years), with less than half female (45.7%). Their racial and ethnic identities were 29.8% White, 31.4% Black, 18.2% Asian, 12.7% Hispanic, and 6.1% Middle Eastern or North African. The countries of origin most represented were the Democratic Republic of Congo (19.4%), Ukraine (17.1%), and Burma (13.2%). Most refugees were married (58.1%), had completed high school (25.3%) or greater than high school (17.9%), had poor English proficiency (62.8%), and were employed (76.0%).

About 37% reported living in a refugee camp. Of those who reported living in a refugee camp, 88% lived in a refugee camp for a year or more or their whole life. Bivariate analyses showed that age, gender, country of origin, race/ethnicity, highest education at arrival, English language proficiency, year of arrival, and resettlement region were significantly associated with living in a refugee camp (Table 2).

**Table 2:** Sociodemographic characteristics of the US refugees by refugee camp living status, ASR 2021-2022.

Characteristic	Did not live in a refugee camp (N = 1,884)^*a*^	Lived in a refugee camp (N = 1,118)^*b*^	p-value^*2*^
**Survey year**			0.019
2021	973 (52%)	528 (47%)	
2022	911 (48%)	590 (53%)	
**Age**	40 (13)	38 (11)	<0.001
**Gender**			<0.001
Female	906 (48%)	467 (42%)	
Male	978 (52%)	648 (58%)	
**Country of origin**			<0.001
Afghanistan	78 (4.2%)	13 (1.4%)	
Burma	198 (11%)	171 (18%)	
Colombia	60 (3.2%)	26 (2.7%)	
Dem. Rep of Congo	132 (7.1%)	411 (43%)	
El Salvador	220 (12%)	22 (2.3%)	
Guatemala	49 (2.7%)	21 (2.2%)	
Iran	46 (2.5%)	14 (1.5%)	
Iraq	132 (7.1%)	9 (0.9%)	
Somalia	44 (2.4%)	60 (6.3%)	
Syria	125 (6.8%)	32 (3.4%)	
Ukraine	477 (26%)	3 (0.3%)	
Other	286 (15%)	170 (18%)	
**Race/ethnicity**			<0.001
Asian	267 (14%)	272 (25%)	
Black	264 (14%)	665 (61%)	
Hispanic	320 (17%)	57 (5.2%)	
Middle Eastern or North African	133 (7.1%)	44 (4.0%)	
Mixed	36 (1.9%)	5 (0.5%)	
White	838 (45%)	44 (4.0%)	
Other	6 (0.3%)	8 (0.7%)	
**Marital status**			0.090
Currently married	1,107 (59%)	611 (56%)	
Not currently married	760 (41%)	478 (44%)	
**Highest education at arrival**			<0.001
None	133 (7.1%)	174 (16%)	
Elementary school	189 (10%)	245 (22%)	
Middle school	338 (18%)	168 (15%)	
High school	432 (23%)	324 (29%)	
Higher	432 (23%)	103 (9.3%)	
Other	354 (19%)	98 (8.8%)	
**Current English language proficiency**			0.001
Very well/Well	737 (39%)	373 (33%)	
Not well/Not at all	1,136 (61%)	744 (67%)	
**Year of arrival**			0.002
2016	235 (13%)	123 (11%)	
2017	339 (18%)	217 (20%)	
2018	245 (13%)	145 (13%)	
2019	411 (22%)	276 (25%)	
2020	383 (20%)	165 (15%)	
2021	265 (14%)	180 (16%)	
**Employment status**			0.2
Employed	1,360 (75%)	856 (77%)	
Unemployed/Not in labor force	450 (25%)	252 (23%)	
**Resettlement region**			<0.001
Midwest	314 (17%)	366 (33%)	
Northeast	321 (17%)	175 (16%)	
South	549 (29%)	359 (32%)	
West	685 (37%)	206 (19%)	

^a^n (%); Mean (SD).

^b^Pearson’s Chi-squared test; Welch Two Sample t-test.

**Fig 1:**
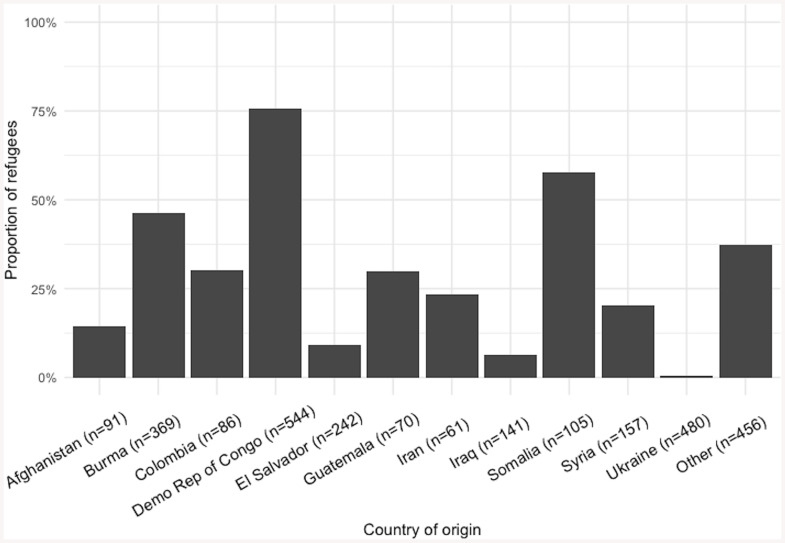
Proportion of refugees in the United States who ever lived in a refugee camp by their country of origin.

The highest proportion of refugees who reported living in a refugee camp were Democratic Republic of Congo (75.7%), Somalia (57.7%), and Burma (46.3%) (Fig 1).

### Major findings

In unadjusted models, refugees who lived in a camp had 17% greater odds of poor physical health and 74% greater odds of poor mental health compared to those who did not live in a refugee camp. Specifically, those who lived in a refugee camp for a year or more had higher odds of poor physical and mental health.

In multivariable adjusted models, having lived in a refugee camp for a year or more was significantly associated with 27% greater odds of poor physical health (aOR: 1.27 [95% CI: 1.02, 1.60]), but the associations with mental health became non-significant at the 0.05 level of significance (Table 3).

**Table 3:** Odds ratios for poor physical and mental health associated with living in a refugee camp, ASR 2021-2022 (N = 3,005).

	Poor physical health		Poor mental health	
**Unadjusted**	**Adjusted** ^**a**^	**Unadjusted**	**Adjusted** ^**a**^
**Variable**	**OR (95% CI); p-value**	**OR (95% CI); p-value**	**OR (95% CI); p-value**	**OR (95% CI); p-value**
**Lived in camp**				
**No**	Reference	Reference	Reference	Reference
**Yes**	1.17 [1.10, 1.37]; 0.05	1.21 [0.98, 1.50]; 0.08	1.74 [1.49, 2.03]; < 0.001	1.11 [0.92, 1.35]; 0.285
**Duration of living in camp**				
Did not live in camp	Reference	Reference	Reference	Reference
Lived in camp: Less than a year	0.96 [0.63, 1.46]; 0.848	0.89 [0.55, 1.45]; 0.652	1.61 [1.08, 2.38]; 0.018	1.23 [0.80, 1.89]; 0.335
Lived in camp: A year or more	1.20 [1.02, 1.41]; 0.031	1.27 [1.02, 1.60]; 0.036	1.76 [1.50, 2.06]; < 0.001	1.09 [0.89, 1.34]; 0.408

OR = Odds Ratio, CI = Confidence Interval

^a^Adjusted for age, gender, country of origin, marital status, highest education at arrival, current English proficiency, year of arrival, employment status, resettlement region at arrival, and survey year.

Full model results are presented in the appendix (S2 Table).

## Discussion

Our study examined the association of living in refugee camps, and duration, with the physical and mental health of refugees resettled in the U.S. In this national sample, we found that slightly over a third of resettled refugees had lived in a refugee camp, and among those, nearly 9 out of 10 had lived in a refugee camp for a year or more of their lives. We found that those who lived in refugee camps had significantly higher odds of both poor physical and mental health, with the association on physical health persisting after adjustment for covariates that included sociodemographic characteristics. This suggests that factors like educational attainment, marital status, and employment status can potentially mitigate mental health harms associated with having lived in a refugee camp. There also appeared to be a dose-dependent relationship, such that those who lived in a refugee camp for a year or more had higher odds of poor physical and mental health, suggesting a cumulative impact over time. Our findings broadly align with previous research that have linked forcible displacement to worse physical health outcomes, when comparing to locals in host countries [21,22], and in pooled data from various countries [23], although these prior studies do not routinely capture refugee camp living as a specific contributing factor, which our findings add to the literature.

Poor physical health outcomes of refugees in camps have been observed in the past and may be associated with refugee camp conditions and the environment. There is an increased risk of infectious diseases due to overcrowding, poor hygiene, and scarce sanitation [24]. Scarcity of access to nutritious food and food insecurity could lead to malnutrition and weakened immune systems, further contributing to physical health deterioration among refugees living in camps [25,26]. Housing challenges and problems accessing affordable housing that is suitable for facing harsh weather conditions may also contribute to poor physical health, especially if linked to respiratory illnesses and other health conditions [27]. Another explanation of poor physical health is attributed to social and structural challenges refugees face in their host countries, like limited access to medical facilities and healthcare, insurance, language barriers, and discrimination [28–30].

The association we found between living in a refugee camp and mental health suggests that other factors may play a more prominent role in influencing mental well-being among refugees. The experience of resettlement itself may have a greater impact on mental health than having been in a camp, with other research pointing to post-resettlement factors like financial instability, employment, and limited social support as contributing to emotional distress [22,31]. In one study of Rohingya refugees living in Bangladesh, post-resettlement employment and humanitarian support was associated with reduced post-traumatic stress symptoms more so than pre-displacement abuse [32]. Stress and psychological trauma are prevalent during the displacement continuum. The stressors during the pre-migration phase include exposure to violence, loss, and persecution [33], while the post-migration phase exposes refugees to additional stressors including acculturation, racism, and discrimination [34], language, loss of family and familiar social support, and the feeling of being extracted from one’s roots [35]. The migration process of each refugee is a multifaceted web of challenges that significantly impact refugees’ mental health, potentially dominating the impact of living in a camp alone. For instance, refugees who have experienced various types of traumas before entering a refugee camp may be more vulnerable to mental health issues regardless of their living situation. Similarly, the stress of cultural adaptation (sometimes dubbed “acculturative stress”), facing discrimination, and family conflict can exacerbate pre-existing vulnerabilities and contribute to increased mental health conditions [33,36]. Since previous literature has shown associations between living in a refugee camp and its conditions and mental health outcomes [8,37], which we did not see when accounting for other sociodemographic variables, future research should certainly investigate specific mediation factors affecting this relationship to inform interventions.

The study findings highlight the urgent and unmet need for targeted interventions addressing the complexity and uniqueness of refugees’ experiences living in refugee camps. Governments and resettlement agencies could prioritize comprehensive, trauma-informed, and culturally sensitive healthcare services for newly arrived refugees with a history of refugee camp residence. Healthcare practitioners could incorporate asking about camp residence in their migration social history to better understand the experiences across the displacement continuum that may impact refugees' health. Importantly, given our finding of greater odds of poor health outcomes among those who have lived in camps for over a year, questions about refugee camp residence should not be a simple yes-no binary but inquire about duration to better understand potential cumulative harm. Future research needs to focus on longitudinal designs to examine the long-term effects of living in refugee camps on physical and mental health and the causal relationship with camp residency duration. Mediating and moderating pre- and post-migration stressors such as employment or host country language proficiency also need to be considered in understanding the influence of living in refugee camps and health outcomes to ultimately plan and implement tailored interventions to improve health outcomes for refugees. Community-based participatory approaches may help design effective, culturally sensitive interventions. In addition, refugee health assessments and surveillance tools could include standardized measures of displacement history, including camp experience, to inform policy and service delivery.

### Strengths and limitations

This study leveraged a large, national sample of recently resettled U.S. refugees to examine the association between refugee camp residence and both physical and mental health outcomes. We examined the health effects of both living in camps as well as duration of camp residence. Additionally, we used multiple imputations to address missing data and adjusted for key sociodemographic factors, strengthening the robustness of our findings. But the study has some limitations. The cross-sectional design limited our ability to establish causality. We also relied on self-report measures, which could have introduced recall bias and cultural differences in health perceptions among participants. Finally, focusing on principal applications only in the inclusion could limit the generalizability of the findings to other family members in refugee camps.

## Conclusions

Residing in refugee camps is significantly associated with worse physical health among recently resettled refugees in the U.S., especially among those who have lived in camps for over a year. These results underscore the importance of policies that minimize prolonged stays in refugee camps and promote timely integration to support refugee health.

## Supporting information

S1 Table Socio-demographic characteristics of the missing versus non-missing sample. (DOCX)

S2 Table Odds ratios for poor physical and mental health associated with living in a refugee camp (full model results), ASR 2021–2022 (N = 3,005). (DOCX)
