# Supplementary material for: Associations between refugee camp living and duration lived in refugee camps with health outcomes: A cross-sectional analysis of the Annual Survey of Refugees, 2021–2022
Source: PLoS One. 2025 Dec 8;20(12):e0327608. doi: 10.1371/journal.pone.0327608 (PMC12685216; doi:10.1371/journal.pone.0327608)
Supplement: S1 Table — (DOCX) [file pone.0327608.s001.docx]

**Supporting information file**

**S1 Table. Socio-demographic characteristics of the missing versus non-missing sample**

| **Characteristic** | **Not missing**  **(N = 2,409)*^1^*** | **Missing**  **(N = 596)*^1^*** | **p-value*^2^*** |
| --- | --- | --- | --- |
| Age | 38 (12) | 42 (15) | <0.001 |
| (Missing) | 0 | 91 |  |
| Gender |  |  | 0.15 |
| Male | 1,323 (55%) | 306 (52%) |  |
| Female | 1,086 (45%) | 287 (48%) |  |
| (Missing) | 0 | 3 |  |
| Race/ethnicity |  |  | <0.001 |
| Asian | 437 (18%) | 102 (18%) |  |
| Black | 641 (27%) | 290 (52%) |  |
| Hispanic | 364 (15%) | 13 (2.4%) |  |
| Middle Eastern or North African | 159 (6.6%) | 19 (3.4%) |  |
| Mixed | 35 (1.5%) | 6 (1.1%) |  |
| White | 762 (32%) | 120 (22%) |  |
| Other | 11 (0.5%) | 3 (0.5%) |  |
| (Missing) | 0 | 43 |  |
| Highest education at arrival |  |  | <0.001 |
| None | 196 (8.1%) | 112 (19%) |  |
| Elementary school | 334 (14%) | 100 (17%) |  |
| Middle school | 409 (17%) | 97 (17%) |  |
| High school | 619 (26%) | 138 (24%) |  |
| Higher | 481 (20%) | 55 (9.4%) |  |
| Other | 370 (15%) | 82 (14%) |  |
| (Missing) | 0 | 12 |  |
| Current English language proficiency |  |  | <0.001 |
| Very well/Well | 939 (39%) | 173 (30%) |  |
| Not well/Not at all | 1,470 (61%) | 411 (70%) |  |
| (Missing) | 0 | 12 |  |
| Employment status |  |  | 0.13 |
| Employed | 1,843 (77%) | 375 (73%) |  |
| Unemployed/Not in labor force | 566 (23%) | 136 (27%) |  |
| (Missing) | 0 | 85 |  |
| *^1^*Mean (SD); n (%) | | | |
| *^2^*Wilcoxon rank sum test; Pearson's Chi-squared test | | | |

**S2 Table. Odds ratios for poor physical and mental health associated with living in a refugee camp (full model results), ASR 2021-2022 (N=3,005)**

|  | **Poor physical health** | | **Poor mental health** | |
| --- | --- | --- | --- | --- |
|  | **Unadjusted** | **Adjusted^a^** | **Unadjusted** | **Adjusted^a^** |
| **Variable** | **OR (95% CI); p-value** | **OR (95% CI); p-value** | **OR (95% CI); p-value** | **OR (95% CI); p-value** |
| **Lived in camp** |  |  |  |  |
| **No** | Reference | Reference | Reference | Reference |
| **Yes** | 1.17 [1.10, 1.37]; 0.05 | 1.21 [0.98, 1.50]; 0.08 | 1.74 [1.49, 2.03]; <0.001 | 1.11 [0.92, 1.35]; 0.285 |
| **Duration of living in camp** |  |  |  |  |
| Did not live in camp | Reference | Reference | Reference | Reference |
| Lived in camp: Less than a year | 0.96 [0.63, 1.46]; 0.848 | 0.89 [0.55, 1.45]; 0.652 | 1.61 [1.08, 2.38]; 0.018 | 1.23 [0.80, 1.89]; 0.335 |
| Lived in camp: A year or more | 1.20 [1.02, 1.41]; 0.031 | 1.27 [1.02, 1.60]; 0.036 | 1.76 [1.50, 2.06]; <0.001 | 1.09 [0.89, 1.34]; 0.408 |
| **Survey year** |  |  |  |  |
| 2021 | Reference | Reference | Reference | Reference |
| 2022 | 1.08 [0.93, 1.26]; 0.306 | 1.03 [0.83, 1.28]; 0.768 | 1.13 [0.97, 1.31]; 0.117 | 0.93 [0.76, 1.14]; 0.513 |
| **Age** | 1.06 [1.05, 1.07]; <0.001 | 1.06 [1.05, 1.06]; <0.001 | 1.00 [1.00, 1.01]; <0.001 | 1.01 [1.00, 1.02]; 0.003 |
| **Gender** |  |  |  |  |
| Male | Reference | Reference | Reference | Reference |
| Female | 1.94 [1.67, 2.27]; <0.001 | 1.76 [1.45, 2.13]; <0.001 | 0.90 [0.77, 1.04]; 0.158 | 0.95 [0.79, 1.15]; 0.611 |
| **Country of origin** |  |  |  |  |
| Other | Reference: | Reference | Reference | Reference |
| Afghanistan | 0.72 [0.44, 1.19]; 0.198 | 0.98 [0.56, 1.71]; 0.937 | 1.01 [0.64, 1.60]; 0.956 | 1.04 [0.64, 1.68]; 0.878 |
| Burma | 1.32 [1.00, 1.76]; 0.048 | 1.37 [0.98, 1.92]; 0.064 | 0.76 [0.57, 1.02]; 0.071 | 0.71 [0.51, 0.97]; 0.033 |
| Colombia | 0.61 [0.36, 1.03]; 0.065 | 0.52 [0.29, 0.93]; 0.028 | 1.40 [0.88, 2.22]; 0.152 | 1.15 [0.71, 1.87]; 0.558 |
| Demo Rep of Congo | 0.90 [0.69, 1.16]; 0.408 | 1.00 [0.73, 1.37]; 0.995 | 2.18 [1.69, 2.81]; <0.001 | 2.00 [1.51, 2.65]; <0.001 |
| El Salvador | 0.38 [0.26, 0.56]; <0.001 | 0.50 [0.32, 0.78]; 0.002 | 0.61 [0.44, 0.86]; 0.005 | 0.61 [0.42, 0.87]; 0.007 |
| Guatemala | 0.84 [0.49, 1.45]; 0.537 | 1.06 [0.57, 1.98]; 0.857 | 0.71 [0.41, 1.24]; 0.234 | 0.67 [0.38, 1.20]; 0.175 |
| Iran | 0.69; [0.39, 1.23]; 0.204 | 0.93 [0.47, 1.83]; 0.832 | 1.16 [0.68, 2.00]; 0.584 | 1.18 [0.67, 2.09]; 0.565 |
| Iraq | 1.54 [1.05, 2.25]; 0.027 | 1.70 [1.09, 2.67]; 0.020 | 1.20 [0.82, 1.76]; 0.358 | 1.23 [0.82, 1.85]; 0.313 |
| Somalia | 0.40 [0.23, 0.68]; <0.001 | 0.41 [0.22, 0.76]; 0.004 | 0.42 [0.25, 0.71]; 0.001 | 0.38 [0.22, 0.66]; <0.001 |
| Syria | 1.54 [1.07, 2.22]; 0.021 | 1.48 [0.96, 2.27]; 0.073 | 2.10 [1.46, 3.03]; <0.001 | 1.86 [1.26, 2.75]; 0.002 |
| Ukraine | 0.82 [0.63, 1.08]; 0.157 | 0.79 [0.56, 1.10]; 0.162 | 0.27 [0.20, 0.37]; <0.001 | 0.30 [0.21, 0.43]; <0.001 |
| **Marital status** |  |  |  |  |
| Not currently married | Reference | Reference | Reference | Reference |
| Currently married | 1.04 [0.89, 1.22]; 0.602 | 0.74 [0.61, 0.90]; 0.002 | 0.78 [0.67, 0.91]; 0.002 | 0.81 [0.68, 0.96]; 0.016 |
| **Highest education at arrival** |  |  |  |  |
| None |  | Reference | Reference | Reference |
| Elementary school | 0.78 [0.58, 1.05]; 0.100 | 1.23 [0.87, 1.73]; 0.236 | 1.29 [0.95, 1.74]; 0.100 | 1.26 [0.91, 1.74]; 0.170 |
| Middle school | 0.59 [0.44, 0.78]; <0.001 | 1.05 [0.74, 1.48]; 0.795 | 0.97 [0.72, 1.30]; 0.814 | 1.30 [0.93, 1.81]; 0.123 |
| High school | 0.31 [0.24, 0.42]; <0.001 | 0.72 [0.51, 1.01]; 0.056 | 0.96 [0.73, 1.26]; 0.754 | 1.15 [0.84, 1.57]; 0.395 |
| Higher | 0.30 [0.22, 0.41]; <0.001 | 0.70 [0.48, 1.02]; 0.066 | 0.68 [0.51, 0.92]; 0.012 | 0.99 [0.69, 1.41]; 0.958 |
| Other | 0.57 [0.42, 0.76]; <0.001 | 0.96 [0.66, 1.40]; 0.827 | 0.53 [0.38, 0.72]; <0.001 | 0.86 [0.60, 1.25]; 0.438 |
| **Current English proficiency** |  |  |  |  |
| Very well/Well | Reference | Reference | Reference | Reference |
| Not well/Not at all | 2.85 [2.40, 3.39]; <0.001 | 1.86 [1.49, 2.32]; <0.001 | 1.24 [1.06, 1.45]; 0.008 | 1.29 [1.06, 1.58]; 0.011 |
| **Year of arrival** |  |  |  |  |
| 2016 | Reference | Reference | Reference | Reference |
| 2017 | 0.98 [0.74, 1.29]; 0.864 | 1.18 [0.83, 1.69]; 0.353 | 0.93 [0.71, 1.22]; 0.595 | 1.06 [0.77, 1.46]; 0.737 |
| 2018 | 0.77 [0.56, 1.05]; 0.098 | 1.04 [0.71, 1.53]; 0.836 | 0.79 [0.59, 1.07]; 0.131 | 0.88 [0.62, 1.25]; 0.481 |
| 2019 | 1.03 [0.79, 1.35]; 0.837 | 1.11 [0.79, 1.55]; 0.558 | 0.86 [0.66, 1.12]; 0.269 | 0.95 [0.70, 1.29]; 0.749 |
| 2020 | 0.89 [0.67, 1.18]; 0.410 | 1.05 [0.75, 1.49]; 0.764 | 0.70 [0.53, 0.93]; 0.014 | 0.83 [0.60, 1.14]; 0.248 |
| 2021 | 1.13 [0.84, 1.51]; 0.413 | 1.34 [0.90, 2.00]; 0.156 | 1.20 [0.90, 1.60]; 0.202 | 1.05 [0.72, 1.51]; 0.811 |
| **Employment status** |  |  |  |  |
| Employed | Reference | Reference | Reference | Reference |
| Unemployed/Not in labor force | 3.31 [2.78, 3.93]; <0.001 | 1.96 [1.59, 2.42]; <0.001 | 0.88 [0.74, 1.06]; 0.172 | 0.90 [0.73, 1.12]; 0.353 |
| **Resettlement region** |  |  |  |  |
| Midwest | Reference | Reference | Reference | Reference |
| Northeast | 0.98 [0.77, 1.25]; 0.869 | 1.01 [0.76, 1.34]; 0.958 | 1.22 [0.96, 1.54]; 0.100 | 1.41 [1.09, 1.82]; 0.009 |
| South | 0.85 [0.69, 1.05]; 0.128 | 1.02 [0.79, 1.30]; 0.902 | 1.00 [0.82, 1.23]; 0.975 | 1.02 [0.81, 1.27]; 0.884 |
| West | 0.84 [0.68, 1.04]; 0.114 | 0.96 [0.74, 1.25]; 0.780 | 0.62 [0.50, 0.77]; <0.001 | 0.83 [0.66, 1.06]; 0.138 |
